# Recurrent bleeding after rubber band ligation diagnosed as mild hemophilia B: a case report and literature review

**DOI:** 10.1186/s12893-022-01553-8

**Published:** 2022-04-01

**Authors:** Xiaoying Jiang, Min Xu, Yaqing Ding, Yongqing Cao, Yibin Pan

**Affiliations:** 1 Clinical Medical College, Longhua Hospital, Shanghai University of Traditional Chinese Medicine, Shanghai, China; 2 Department of Hematology, Longhua Hospital, Shanghai University of Traditional Chinese Medicine, Shanghai, China; 3 Department of Anal-Rectal Surgery, Longhua Hospital, Shanghai University of Traditional Chinese Medicine, 725 WanPing South Road, Shanghai, China

**Keywords:** Postoperative bleeding, Surgery, Coagulation factors, Prothrombin complex

## Abstract

**Background:**

Hemophilia is a recessive hemorrhagic disease relevant to X chromosome. In mild hemophilia cases, spontaneous bleeding is rare and the blood clotting function is normal, but severe bleeding may occur after trauma or surgery. Therefore, missed diagnosis of hemophilia before operation may contribute to bleeding after hemorrhoid operation.

**Case presentation:**

A 21-year-old male was hospitalized in the anorectal department because of repeated bleeding after hemorrhoid surgery. Despite several suture hemostasis procedures, the patient still suffered from recurrent bleeding. He had no family history of hemophilia or bleeding tendency, and had not been diagnosed with hemophilia before this admission. The diagnosis of mild hemophilia B was made after further examination of coagulation indexes. By using frozen plasma and coagulation factor complex to supplement coagulation factors, the patient’s bleeding was stopped and he was discharged after 23 days in hospital. During the follow-up, lower-than-normal coagulation factors were still found in him, but no bleeding occurred again.

**Conclusions:**

The undiagnosed patient with mild hemophilia B has an increased risk of bleeding after hemorrhoid surgery because of the consumption of coagulation factors. This case report aims to address the importance of hemophilia screening before operation and reduce the risk of postoperative bleeding. For patients with recurrent bleeding after hemorrhoid surgery, hemophilia should be further excluded. Wound bleeding may recur in hemophilia patients after suture hemostasis. Therefore, prompt supplementation of coagulation factors is needed to help stop bleeding once the diagnosis of hemophilia is made.

## Background

Hemophilia, which includes hemophilia A and hemophilia B, is a recessive hemorrhagic disease linked with X chromosome. Hemophilia A is caused by the deficiency of coagulation factor VIII (FVIII), while hemophilia B is resulted from the deficiency of coagulation factor IX (FIX). Hemophilia patients present with normal platelet (PLT), prothrombin time (PT), thrombin time (TT), blood clot retraction test (CRT) and bleeding time (BT), and only mildly prolonged activated partial thromboplastin time (APTT), which may lead to missed diagnosis of hemophilia before operation. Here, we report a 21-year-old male with mild hemophilia B who developed recurrent bleeding after rubber band ligation (RBL), and review the previously published cases of bleeding after hemorrhoid surgery.

## Case presentation

A 21-year-old male patient was admitted to the anorectal department of a tertiary hospital for acute and severe recurrent bleeding after RBL. The patient was diagnosed with mixed hemorrhoids (connective tissue external hemorrhoids with internal hemorrhoids of third grade) and underwent RBL on July 2, 2020. Pulsatile bleeding occurred at Day 18 and Day 23 after operation, both resolved by suture hemostasis in a community hospital. And this was the third time of bleeding, 26 days after RBL.

At admission, the patient’s body temperature was 38.0 ℃, heart rate 90, blood pressure 147/75 mmHg, respiratory rate 18, and oxygen saturation 98% under oxygen inhalation. He was weak, dizzy, and sweaty. His abdomen was soft with the bowel sound 7 times/min, and there was no tenderness or rebound pain in palpation. Massive anorectal bleeding was observed and the wound in the operation area had not healed. His blood test showed that hemoglobin was 145 g/L (normal range 130–175 g/L) and PLT was 178 × 10 ^9^/L (normal range 125–350 × 10 ^9^/L). Coagulation study showed that his PT was prolonged to 13.8 s (normal range 9.8–12.1 s) and his FVIII was 117% (normal range 70–150). Leucocyte, biochemical examination, infection markers (AIDS, syphilis, and hepatitis), abdominal ultrasound and chest X-ray examination results were all negative.

**Fig. 1:**
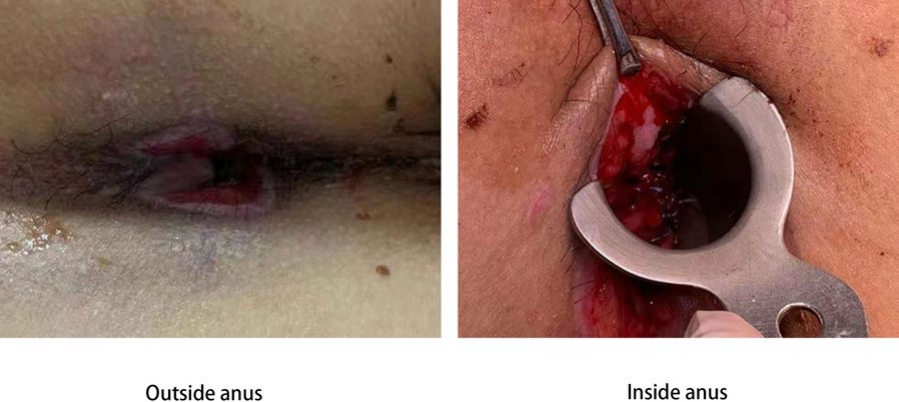
The hemostatic position of suture ligation in the last operation

**Fig. 2:**
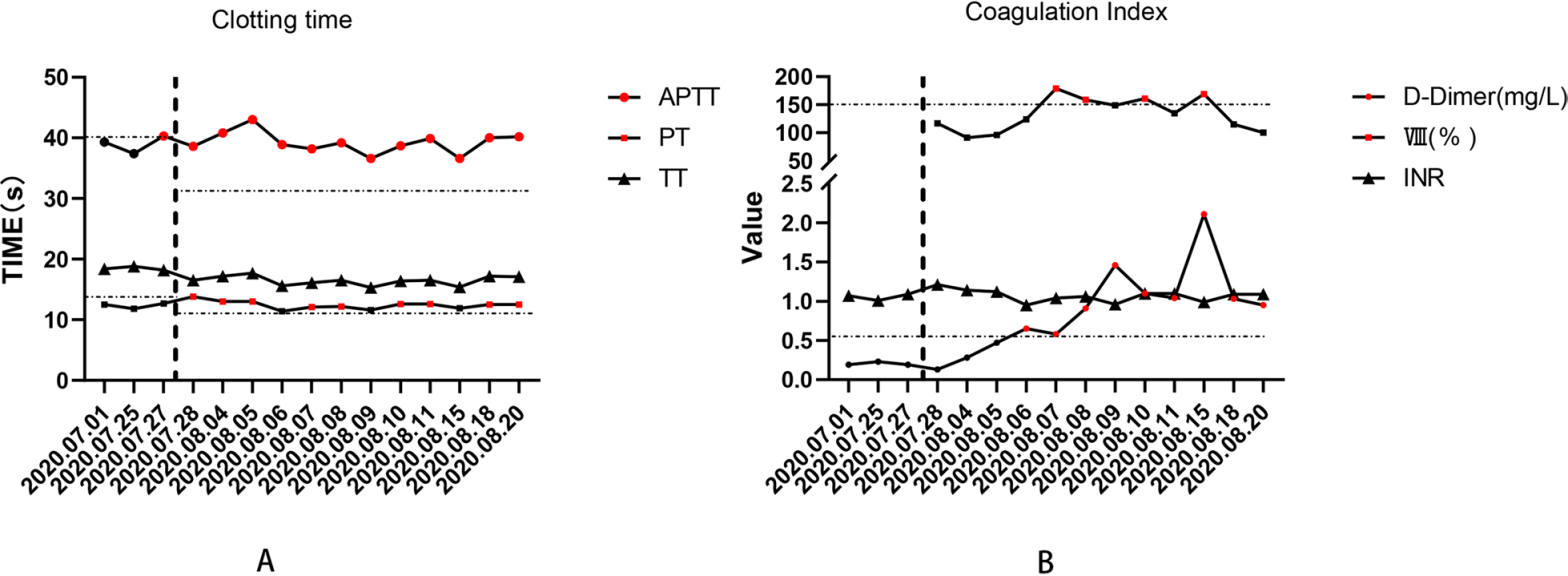
**A** Clotting time; **B** Coagulation index. Notes: The red dots in the picture all indicate higher than normal. Thick dotted lines indicate the point patient was admitted. Thin dotted lines indicate high values in the normal range

**Fig. 3:**
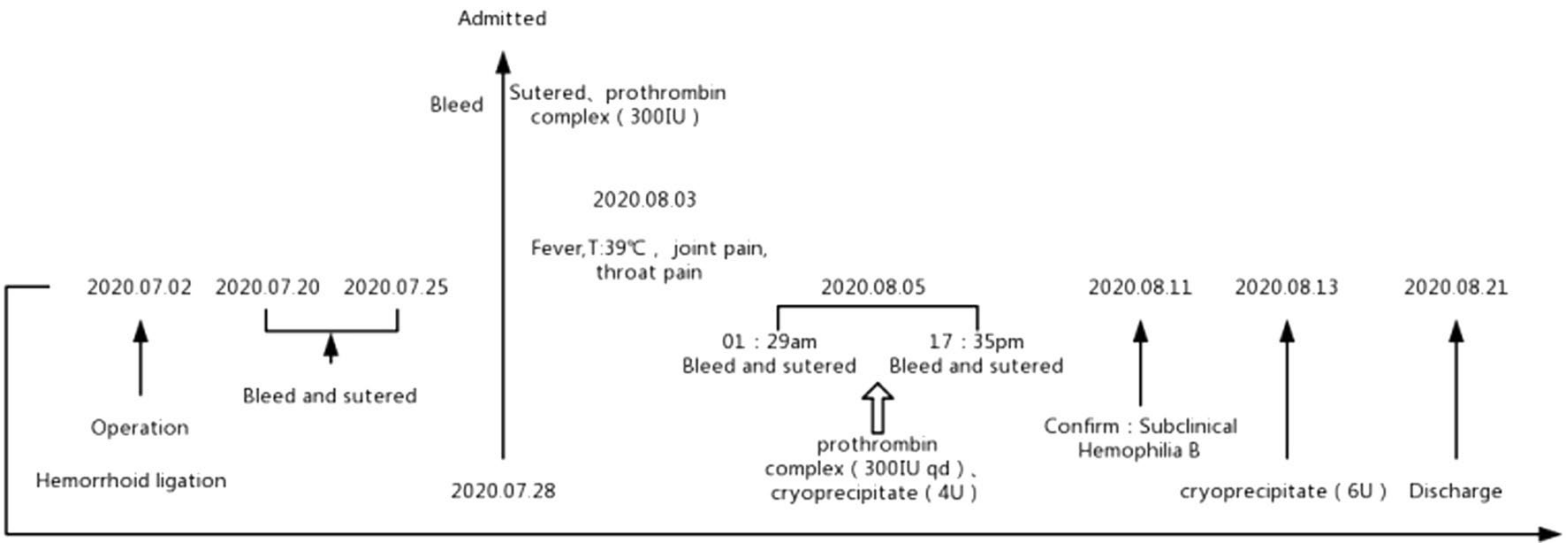
On July 28th, 2020, the patient was hospitalized with postoperative bleeding income and completed suture hemostasis

The patient received patient-controlled analgesia pump (PCA) to control the pain and was sent to the operating room to stop bleeding immediately after admission. Fluctuating bleeding occurred at 11 o’clock and 7 o’clock in the lithotomy position during the operation, which was stopped by suturing. He was given a full liquid diet for 3 days. Human prothrombin complex concentrates (PCC) were given after the operation and maintained for 3 days. At Day 6 after operation, the patient developed joint pain and sore throat with a body temperature of 38–39 ℃. Blood test showed that the percentage of neutrophils was 87.6%. Coagulation study revealed that factor VII (FVII) was 40%, FIX 11%, FX 71.4%, FXII 35%, and PT 16.7 s, APTT 59.2 s. At 1: 30 a.m. Day 8 after operation, the wound bled again. Under anesthesia, mucosal hyperemia and bleeding was found at 7 o’clock and 11 o’clock in the lithotomy position. The patient underwent suture hemostasis in the operating room, and then received 4u cryoprecipitate. At 16:30, bleeding occurred again. During the operation, active bleeding was found at 11 o’clock and blood oozing at 5 and 7 o’clock of the wound in the lithotomy position. The wound was sutured again (Fig. 1). The patient was then strictly forbidden to eat and drink, given total parenteral nutrition (TPN), and treated with the compound of etamsylate and PCC. At Day 14, the patient was diagnosed with mild hemophilia B. PCC (300iu) was precipitated twice a day. There was no further bleeding. During the treatment, immune system examination and DADE'PFA Collage/EPI test were performed to rule out other diseases that might lead to bleeding. The changes of coagulation indexes are presented in Fig. 2A and B. The process of diagnosis and treatment are shown in Fig. 3. At Day 25, the patient was discharged. At the one-month follow-up, his coagulation FIX was 9.1%. The patient’s mother was tested for clotting time after the patient’s discharge, showing negative results. Genetic test was not performed.

### Review

By reviewing literature in Pubmed, we found 5 hemophilia cases with hemorrhoidectomy, including 4 hemophilia A cases and 1 hemophilia B case [1–5] (Table 1). Among them, 3 patients were diagnosed with hemophilia and received basic treatment for hemophilia before the operation. They had blood coagulation factor examination and the blood coagulation factors were supplemented to normal or above normal level preoperatively. After bleeding, their coagulation factors and coagulation factor inhibitors were checked, and coagulation factors were supplemented quickly in large doses. The other two cases had not been diagnosed with hemophilia before operation. One of them who developed repeated bleeding 8 days after the operation was sutured for many times to stop the bleeding, and even received abdominoperinectomy. The other one was diagnosed with hemophilia in the preoperative examination and given epsilon amino caproic acid (EACA).

**Table 1:** Cases in anorectal Department

Number	Author	Gender	Age	Type	First diagnosed	Factor levels	Preoperative factor levels	Postoperative bleeding	Factor level after hemorrhage	Postoperative hemorrhage time	Inhabitors	Therapeutic regimen
1	Orangio et al. (1989) [9]	M	22	A	Y	7%	100%	Y	50%	8d	( +)	Abdominoperinectomy, Surgical hemostasis, PRBC, Cryoprecipitate, FFP, platelet, FVIII
2	Goudemand et al. (2004) [10]	M	52	B	N	/	/	Y	/	8d	( +) (1.3%)	FVII, FIX
3	Beck. et al. (1969) [11]	M	52	A	N	5%	30%	Y	0%	17d	(+)	Cryoprecipitate, steroid
4	Santos-Dias et al. (1992) [12]	M	20	A	N	20%	150%	N	/	/	/	DDAVP
5	Murali et al. (1987) [13]	M	38	A	Y	1%	123%	Y	Null	4d	( +)	FVIII, Cryoprecipitate, PRBC, EACA

FFP, fresh frozen plasma; PRBC,: packed red blood cell; EACA, epsilon amino caproic acid

In addition, the cases of PubMed for 10 years were searched with the MeSHs of “Postoperational Haemorrhage” and “Haemophilia” [6–11] (Table 2). All patients were male and diagnosed for the first time, with no preoperative related coagulation factors were mentioned. The second patient eventually died of fulminant hemorrhagic shock. Due to it was difficult to find a definite bleeding point in the examination after postoperative bleeding, so it was difficult to stop bleeding by surgery. Once these patients received the supplement of coagulation factors and reduce the inhibitors, the bleeding can be alleviated and the wound will eventually heal. This suggests that hemophilia should be taken into account for unexplained and recurrent postoperative bleeding.

**Table 2:** Cases in general surgery

Number	Author	Age	Type	Surgery	Bleeding site	History of operation (related to the bleeding)	Factor level after hemorrhage	Postoperative hemorrhage time	Inhabitors	Therapeutic regimen
1	Asher et al. (2020) [6]	53	AHA	ileal-cecal bypass	Upper digestive tract	12	/	1 month	(+)	superior mesenteric artery embolectomy
2	Mekenkamp, et al. (2015) [7]	67	AHA	pancreaticoduodenectomy	Superior mesenteric artery, Right internal mammary artery	2	8%	7d	(+)	Embolism, RBCs, platelets, FFP, tranexamic, vitamin K, prednisone, cyclophosphamide
3	Hollmig et al. (2014) [8]	86	AHA	Mohs micrographic surgery	Surgical site (left nasofacial sulcus)	1	< 1%	Several hours	(+)	PRBCs, FFP, cryoprecipitate, FVIII; prednisone
4	Onishi et al. (2013) [9]	71	AHA	Cholangiocarcinoma surgery	Intraabdominal	/	1%	5d	(+)	PCC, prednisone, rituximab
5	Plog et al. (2013) [10]	79	AHA	anterior resection	surgical site(drains and wounds)	/	43%	2d		PRBCs, FFP, FVIII
6	Miura et al. (2012) [11]	79	AHA	pancreaticoduodenectomy	Intraabdominal	/	2%	3d	(+)	PRBCs, FFP, PCC, prednisone, cyclophosphamide
7	Miura et al. (2012) [11]	67	(FII, V, VII, IX, X)	hepatectomy	Intraabdominal	/	/	7d	(+)	bovine thrombin, prednisone

FFP, fresh frozen plasma; PRBC, packed red blood cell; EACA, epsilon amino caproic acid; AHA, Acquired hemophilia A

Hemophilia B presents with less severe symptoms compared to hemophilia A, in terms of imaging score of joint diseases, serological indexes, HSS score and joint replacement rate [12–14]. In clinical practice, surgeons tend to pay more attention to the shortening of coagulation time or the screening of hemophilia A during the perioperative examination, and thus ignore the screening of hemophilia B. Yet, hemophilia B can also increase the risk of bleeding in patients undergoing surgery.

Hemophilia patients are classified into three categories according to the activity level of coagulation factors [15]. The level of clotting factors in patients with mild hemophilia is 5–40 IU/dl; their major symptom is severe bleeding following major trauma or surgery, and spontaneous bleeding is rare. The level of clotting factors in patients with moderate hemophilia is 1–5 IU/dl; their symptoms include occasional spontaneous bleeding and prolonged bleeding following minor trauma or surgery. The level of clotting factors in patients with severe hemophilia is < 1 IU/dl; their symptom is spontaneous bleeding into joints or muscles, predominantly in the absence of identifiable hemostatic challenge. It is difficult to detect mild to moderate hemophilia by symptoms before operation. In China’s guidelines [16] for hemophilia patients, hemophilia A patients should be given 80–100 IU/dl factor replacement therapy for the initial treatment and the dosage should be maintained at 50 IU/dl for 7–14 days, and hemophilia B patients should be given 60–80 IU/dl factor replacement therapy for the initial treatment and the dosage should be maintained at 30 IU/dl for 7–14 days. The expected dosage in the perioperative period of minor surgery is 50–80 IU/dl, which should be maintained for 1–5 days according to the type of surgery. The WFH’s [17] surgical recommendations for hemophilia patients state that the coagulation factor level of all patients should be greater than 50 IU/dl before anesthesia, and the preoperative and postoperative evaluation of all hemophilia A and B patients should include inhibitor screening and determination. For patients with mild hemophilia A (no medical contraindication) with slight bleeding or undergoing surgery, DDAVP (1-deamino-8-D-argininevasopressin) may be an effective hemostatic treatment. For hemophilia patients, the surgical treatment should consider the risk of thrombosis, and fibrinolytic drugs and local hemostatic drugs should be used as appropriate. Whether to use factor replacement therapy or not should be determined after intraoperative blood loss evaluation. Intraoperative blood loss of hemophilia patients are divided into four levels: < 10%, 10%–25%, 25% and > 50%. For the first two levels, no extra (unplanned) dose of FVIII/FIX/bypass agent is needed, and the blood component transfusion demand is similar to that in non-hemophilia patients. For the third level, additional (unplanned) doses of FVIII/FIX/bypass or blood components are needed and the expected blood transfusion demand should be increased (within 2 times). For the last level, accidental hypotension or accidental transfer to the intensive care unit due to bleeding should be considered and a significant increase in blood component transfusion demand is expected (> 2 times).

## Discussion and conclusion

In this case, the patient suffered from postoperative bleeding for five times, with intervals of 16 days, 5 days, 5 days, 7 days, and 16 h, respectively. The first bleeding may be caused by post hemorrhoidectomy secondary hemorrhage (PHSH, which usually occurs several days after operation in 0.6% to 5.4% of cases [18]), or delayed cutaneous wound healing resulted from hemophilia B [19]. With the consumption of coagulation factors, the bleeding interval was shortened. Since prothrombin complex was given after this admission, the wound bleeding was stopped for the following 7 days. The interval between the last two bleedings was only 16 h, which may be related to the massive consumption of coagulation factors and the congestion and erosion of mucous membranes after repeated suturing. It was worth noting that the patient developed joint pain on Day 5 after admission, which may be a precursor of bleeding. Due to the lack of preoperative coagulation indexes, it is difficult to determine whether this is secondary hemophilia caused by surgery, so follow-up is needed after discharge. When asked about the medical history, the patient mentioned that he had experienced massive bleeding after oral surgery, making the diagnosis more inclined to congenital hemophilia.

Hemorrhoids are highly prevalent in the general population: 38.9% of the adult population suffer from hemorrhoids, and about 8% of them have grade III or IV hemorrhoids [20]. The evidence of surgical interventions for grade III or IV hemorrhoids are convincing [21–23]. In contrast, the incidence of hemophilia is low (1/5000 for HA, and 1/30000 for HB) [24]. In addition, mild hemophilia could hardly be detected by routine preoperative examination, which increases the risk of delayed healing, repeated bleeding, and massive bleeding after operation. So more specialized examination of coagulation factors should be conducted in the perioperative period. Once hemophilia is diagnosed, exogenous coagulation factors should be actively and pertinently supplemented. Suturing alone can hardly stop the bleeding. And the patient needs close monitoring and follow-up according to the guidelines of hemophilia.

For severe recurrent bleeding, apart from local suture hemostasis, digital subtraction angiography (DSA) is also used by some surgeons to locate the bleeding point or higher blood vessels so as to block the blood supply in the operation area.

In the recent literature, for major operations such as knee arthropathy surgery, it has been proved that only replacement therapy can not reduce the risk of postoperative bleeding, and stronger medical assistance is needed [25]. This may explain why a greater injury in the lower rectum would lead an unsatisfactory of curative effect, even if coagulation factors were supplemented to a normal level. Therefore, it is more important to choose a less invasive surgical method. Combined with 3% polidocanol foam sclerotherapy could reducing the risk of delayed bleeding [26]. And compared with traditional surgical methods, new surgical options such as laser hemorrhoidoplasty [27], arterial embolization [28] and other high-energy devices included ultrasound or radiofrequency [29, 30] have been reported in the literature to be related to less trauma, pain and other postoperative discomfort.

Besides the choice of preoperative surgical methods, drug supplementation and postoperative hemostasis, psychological intervention is also suggested. And patients tend to avoid physical activities due to repeated bleeding, even under sufficient treatment conditions. After the bleeding is stop, some patients still prefer to stay in bed for a long time without any movement, which may increase the risk of deep vein thrombosis and is not conducive to the recovery.

## Data Availability

Data sharing is not applicable to this article, as no datasets were generated or analyzed during the current study.

## Abbreviations

FVIII: Coagulation factor VIII
FIX: Coagulation factor IX
PLT: Normal platelet
PT: Prothrombin time
TT: Thrombin time
CRT: Retraction test
BT: Bleeding time
APTT: Activated partial thromboplastin time
RBL: Rubber band ligation
PCA: Patient-controlled analgesia pump
PCC: Prothrombin complex concentrates
TPN: Total parenteral nutrition
FFP: Fresh frozen plasma
PRBC: Packed red blood cell
EACA: Epsilon amino caproic acid
DDAVP: 1-Deamino-8-D-argininevasopressin
DSA: Digital subtraction angiography
